# Loss of function mutation in toll-like receptor-4 does not offer protection against obesity and insulin resistance induced by a diet high in trans fat in mice

**DOI:** 10.1186/1476-9255-8-2

**Published:** 2011-02-11

**Authors:** Matam Vijay-Kumar, Jesse D Aitken, Frederic A Carvalho, Thomas R Ziegler, Andrew T Gewirtz, Vijay Ganji

**Affiliations:** 1 Pathology and Laboratory Medicine, Emory University School of Medicine, Atlanta, GA 30322, USA; 2 Division of Endocrinology, Metabolism and Lipids, Department of Medicine, Emory University School of Medicine, Atlanta, GA 30322, USA; 3 Division of Nutrition, School of Health Professions, College of Health and Human Sciences, Georgia State University, Atlanta, GA 30302, USA

## Abstract

**Background:**

Toll-like receptor-4 (TLR4) triggers inflammatory signaling in response to microbial lipoploysaccharide. It has been reported that loss of TLR4 protected against saturated fat-induced inflammation and insulin resistance. It is not known whether loss of TLR4 function offers protection against trans fat (TF) induced obesity, inflammation, and insulin resistance. We investigated whether mice with loss of function mutation in TLR4 were resistant to TF-induced pathologies such as obesity, inflammation, hyperglycemia, and hyperinsulinemia.

**Methods:**

C57BL/6j and C57BL/10 mice were cross bred to generate TLR4 mutant and wild type (WT). TLR4 mutant (n = 12) and WT (n = 12) mice were fed either low fat (LF) (13.5% fat energy) or high TF diets (60% fat energy) for 12 weeks. *In vitro *experiments were conducted on mouse macrophage cells (RAW 264.7 and J774A.1) to investigate whether elaidic (trans 18:1) or oleic acid (cis 18:1) would upregulate inflammatory markers.

**Results:**

TLR4 mutant mice were ~26.4% heavier than WT mice. In both genotypes, mice that received TF diet were significantly heavier than those mice that received LF diet (P < 0.01). TLR4 mutant mice compared to WT mice had significantly higher fasting blood glucose, serum insulin, insulin resistance, serum leptin, and serum cholesterol when they received TF diet (P < 0.05). No upregulation of iNOS or COX2 in response to either elaidic or oleic acid in macrophage cells was observed.

**Conclusions:**

Loss of function mutation in TLR4 not only did not protect mice from TF-induced obesity, hyperglycemia, hyperinsulinemia, and hypercholesterolemia but also exacerbated the above pathologies suggesting that functional TLR4 is necessary in attenuating TF-induced deleterious effects. It is likely that TF induces pathologies through pathways independent of TLR4.

## Background

Recently, there has been a considerable interest on the negative health effects of trans fat (TF) in the diet. TFs are unsaturated fats with at least one double bond in the trans configuration. Major dietary sources of TFs are fried foods, bakery products containing hydrogenated vegetable oils and shortenings, margarines, and butter. TFs increase the risk for cardiovascular diseases (CVD) by raising LDL-cholesterol and lipoprotein (a), lowering HDL-cholesterol [1], and adversely affecting the endothelial function [2]. The increased risk of CVD with TF intake is higher than the predicted effects of TF on serum lipids alone suggesting an additional non-lipid role for TF in the pathobiology of CVD [3]. Additionally, epidemiological evidence suggests that increased intake of TF increases the risk of type 2 diabetes [4-6]. The underlying mechanism through which TF influences the development of type 2 diabetes is not clearly understood.

Toll-like receptors (TLRs) are pattern recognition receptors that play a crucial role in mediating the host's innate immune response against microbial products. TLRs are expressed in various human tissues [7]. The specific TLR, TLR4 has been implicated in fatty acid induced inflammation and insulin resistance [8-11]. TLR4 is activated by lipopolysaccharide (LPS), a bacterial endotoxin produced by Gram negative bacteria. The binding of LPS with TLR4 is complex and involves CD-14 and the adaptor proteins, MD-2 and myeloid differentiation factor-88 (MyD88). The process recruits interleukin-1 receptor-associated kinase (IRAK) leading to the activation of nuclear factor kappa B (NF-κB) [12,13]. This activated NF-κB translocates into the nucleus and induces the production of inflammatory cytokines [14].

Recently, several investigators have proposed that saturated fatty acids act as agonists for TLR4. Observations from these studies suggested that the saturated fat-induced TLR4 signaling pathway is a likely mechanism linking dietary fat with inflammation and insulin resistance associated with obesity and type-2 diabetes [9,10,15-21]. Conversely, loss of function of TLR4 led to the protection of saturated fat-induced insulin resistance and diet-induced obesity in mice [8,22,23]. Given the broad spectrum of agonists for TLR4, it is possible that TLR4 may have much broader role than is currently known [18].

Given the similarities between saturated fat and TF in raising blood cholesterol and transducing the production of inflammatory markers, we hypothesized that TF is a potential agonist for the TLR4. Further, we hypothesize that a loss of function mutation in TLR4 may protect against high TF diet-induced inflammation and insulin resistance in mice. Therefore, the objective of this current study was to investigate whether loss of function mutation in TLR4 protects against TF rich diet-induced inflammation and insulin resistance in a mouse model.

## Methods

### Diets and Reagents

Rodent diet high in TF (Catalog #D06061202) from Research Diets Inc (New Brunswick, NJ) and rodent regular low-fat (LF) chow (control diet) (Catalog #5001) from Lab Diets, Inc were purchased. Nutrient composition of the LF control diet and the high TF diet are presented in Table 1. The high TF diet provided 60% of total dietary energy from fat (34.9 g/100 g) with TF accounting for 65.4% of the total fat content. The source of TF was shortening. The LF diet provided 13.5% energy from fat. The LF diet was trans fat free.

**Table 1:** Composition of laboratory diets

Ingredient	Low-fat (control) diet g/100 g^1^	High-trans fat diet g/100 g^2^
Protein	23.9	-
Casein	-	25.8
L-cystine	-	0.4
Carbohydrate	48.7	-
Maltodextrin	-	16.2
Sucrose	-	8.9
Fiber	5.1 (crude)	-
Cellulose	-	6.5
Fat		
Sunflower oil	5.7	-
Soybean oil	-	3.2
Shortenning^3^	-	31.7
Ash	7.0	-
Mineral mix	-	1.3
Vitamin mix	0.25	1.3
Energy, *kj/g*	17.1	21.9
Protein, *% energy*	28.5	20.0
Carbohydrate, *% energy*	58.0	20.0
Fat, % energy	13.5	60.0

^1^Non-purified diet

^2^Purified diet (trans fat free)

^3^Primex shortening containing 65.4 g of trans fat/100 g of total fat

Oleic and elaidic acids were purchased from Matreya (Pleasant Gap, PA). LPS and bovine serum albumin (BSA) (fatty acid free and low endotoxin) purchased from Sigma (St Louis, MO). Rabbit anti-mouse cyclooxygenase-2 (COX-2) was purchased from Cayman Chemical Co (Ann Arbor, MI). Rabbit anti-mouse inducible nitric oxide synthase (iNOS) was purchased from Upstate (Lake Placid, NY). Donkey anti-rabbit IgG antibodies conjugated with horseradish peroxidase were purchased from GE Health Care (Piscataway, NJ). All other reagents were purchased from Sigma.

### Cell culture

Mouse macrophage cell lines RAW 264.7 and J774A.1 cells were cultured in Dulbecco's modified Eagle's medium containing 10% (v/v) heat-inactivated fetal bovine serum albumin (BSA), 100 units/ml penicillin, and 100 μg/ml streptomycin at 37°C in a 5% CO_2_. Cells (5 × 10^5^) were plated in 6 well plates and cultured in the presence of LPS (20 ng/ml), elaidic acid-BSA, or oleic acid-BSA complexes for 24 h [17]. Supernatants and cell lysates were stored at -80°C until analyzed.

### Mice

C57BL6/j and C57BL/10 mice were purchased from Jackson Laboratories (Bar Harbor, ME) and cross bred to generate wild type (WT) mice and mice with loss of function mutation in TLR4. C57BL/6j is the most commonly used background strain for the generation of transgenic mice in research areas such as diabetes, obesity, and immunology because this strain is susceptible to diet-induced obesity and type-2 diabetes. Also, this strain breeds well, has longer life span, and is less susceptible to tumors. C57BL/10 is a homozygous strain for TLR4 mutation. Genotyping was performed on all experimental mice before the study. Genotyping of TLR4 mutant and WT mice were confirmed with PCR. Mice were genotyped using tail DNA extracted with Sigma RedExtract kit from Invitrogen (Carlsbad, CA).

Animals were housed at 22°C with an automatically controlled 12-h light and dark cycles. A total of 24 male mice (12 WT and 12 with the loss of function mutation in TLR4) of 4-wk old were used. Within each genotype, 6 mice received a control LF diet and 6 mice received a high TF diet for 12 weeks. Mice had unlimited access to diets and water. During the course of feeding, food intakes were monitored on a daily basis. All experimental protocols involving animals were approved by the Animal Ethical Committee at Emory University and Georgia State University, Atlanta, GA.

### Tissue harvest and liver histology

After collecting blood using the cardiac puncture procedure, liver, cecum, and epididymal fat tissues were separated using sterile equipment and weighed. Liver tissue was placed in 4% paraformaldehyde and embedded in paraffin. Tissue sections of 5 μm in thickness were stained with hematoxylin and eosin and pictures were taken with a microscope that was fitted with a camera.

### Measurement of biochemical parameters

At the end of the 12 week feeding period, mice were fasted for 5 h and blood was collected from retrobulbar intraorbital capillary plexus. Serum was generated by centrifugation of blood using serum separator tubes (Becton Dickinson, Franklin Lakes, NJ). Serum was stored after insulin injection at -80°C until analyzed. Serum cholesterol and triglycerides were quantified by colorimetric kits from BioVision (Mountain View, CA). Serum insulin, leptin, and adiponectin were analyzed by ELISA kits purchased from Linco Research Inc (St. Charles, MO). Insulin resistance was calculated from fasting glucose and serum insulin concentrations using homeostatic model assessment (HOMA-IR) (Fasing glucose (mg/dL) × μunits/mL ÷ 450).

### Western blotting

COX-2 and iNOS were measured in macrophage cell lysates by immunobloting as previously described (17). IL1-β and IL-6 concentrations in supernatants were measured using ELISA (R&D Systems, Minneapolis, MN) according to the manufacturer's instructions. β-Actin served as a loading control.

### Data Analysis

Data were presented as mean ± SD of mean. The effect of genotype (WT and TLR4 mutant) within the diet (LF and TF) was assessed with a 2-way analysis of variance (genotype × diet). Group-wise comparisons between WT and TLR4 mutant mice within the LF or TF diet were performed with Bonferroni posttest (GraphPad Prism software, San Diego, CA). Statistical significance was set at *P *< 0.05.

## Results

### Loss of TLR4 did not protect from high TF diet-induced obesity

**Figure 1:**
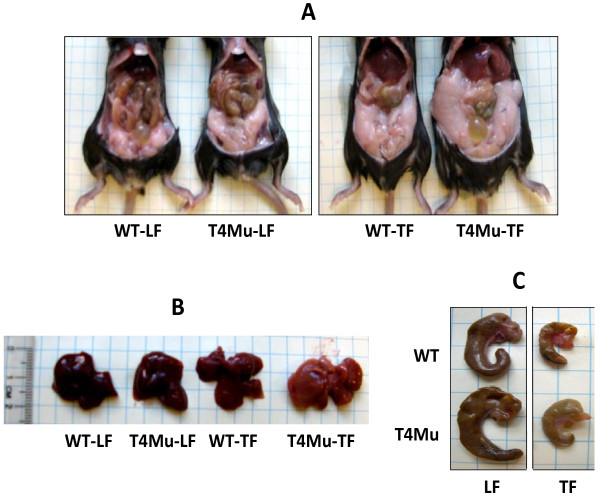
**Morphologies of visceral fat (A), liver (B), and cecum (C) of WT and T4Mu mice that received LF and TF diets**. Abbreviations: LF, low fat diet (13.5% energy from fat); T4Mu, toll-like receptor-4 loss of function mutant mice; TF, trans fat diet (60% of energy from fat, shortening-based); WT, wild type mice.

**Figure 2:**
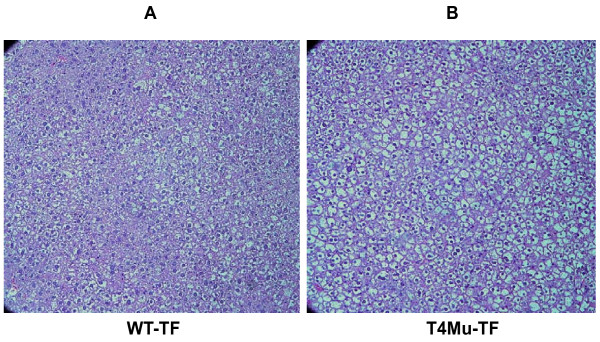
**Liver histology of WT and T4Mu mice that received TF diet**. For simplicity, histology related to mice that received LF diet is not presented as there were no differences. Abbreviations: T4Mu, toll-like receptor-4 loss of function mutant mice; WT, wild type mice.

Average daily food intakes were not significantly different between WT mice and mice with loss of function mutation in TLR4 fed either LF diet (3.2 vs. 3.1 g/d/mouse) or TF diet (2.3 vs. 2.6 g/day/mouse). Visceral fat (fat pad), liver, and cecum morphologies of WT and TLR4 mutant mice are displayed in Figure 1A-C. The appearance of the liver from the TLR4 mutant mice that received TF diets looked paler compared to those mice that received control diet suggesting fat deposition in the liver. Upon further histological examination, TLR4 mutant mice that received TF had more and larger fat vesicles than WT mice that received TF (Figure 2A and 2B).

**Figure 3:**
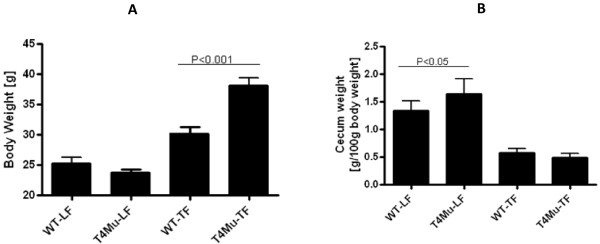
**Body weights (A) and cecum weights (B) of WT and T4Mu mice that received LF and TF diets**. Significant difference was determined with 2-way analysis of variance (genotype × diet type) for body weights and cecum weights. Body weights and cecum weights are significantly higher in T4Mu mice compared to WT mice that received TF diet. Abbreviations: LF, low fat diet (13.5% energy from fat); T4Mu, toll-like receptor-4 loss of function mutant mice; TF, trans fat diet (60% of energy from fat, shortening-based); WT, wild type mice.

After 12 wk of feeding, body weights were significantly higher in mice with loss of function mutation in TLR4 compared to WT mice that received TF diet (30.2 vs. 38.2 g; P < 0.001). Body weights are presented in Figure 3A. On average, TLR4 mutant mice were ~26.4% heavier in comparison to WT mice. Regardless of genotype, as expected, body weights of mice that received the TF diet were significantly higher compared to body weights of those mice that received LF diet (P < 0.01). Weights of visceral fat pad followed similar patterns (data not shown). Weights of the full-thickness cecum were significantly lower in both genotype mice that received TF diet (P < 0.001) (Figure 3B). Liver and spleen weights were not significantly different between genotypes that received either a LF diet or TF diet or between diet types within the genotype (data not shown).

### Serum insulin, blood glucose, insulin resistance, serum adipokines, and selected inflammatory markers

**Figure 4:**
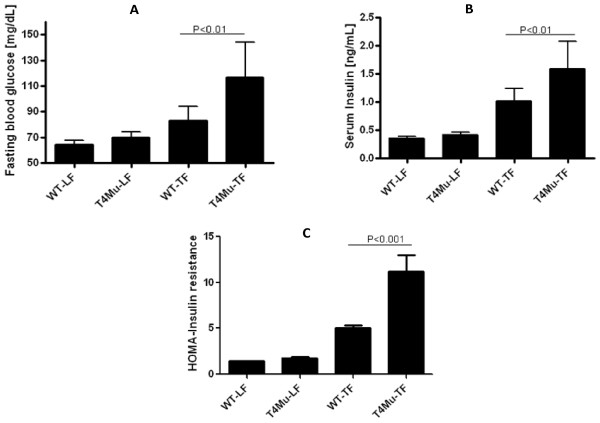
**5-hour fasting blood glucose (A), serum insulin (B), and HOMA-IR of WT and T4Mu mice that received LF and TF diets**. Significant difference was determined with 2-way analysis of variance (genotype × diet type) for blood glucose, serum insulin, and HOMA-IR. Fasting blood glucose, serum insulin, and HOMA-IR are significantly higher in T4Mu mice compared to WT mice that received TF diet. Abbreviations: HOMA-IR, Homeostatic Model Assessment-Insulin Resistance; LF, low fat diet (13.5% energy from fat); T4Mu, toll-like receptor-4 loss of function mutant mice; TF, trans fat diet (60% of energy from fat, shortening-based); WT, wild type mice.

Serum insulin and blood glucose concentrations were measured after 5 h fasting. In both genotypes, mice that received TF diet had higher blood glucose and serum insulin concentrations compared to those that received LF diet. Mice with loss of function mutation in TLR4 compared to WT mice had significantly higher blood glucose (116 vs. 83 mg/dL; P < 0.01) and serum insulin concentrations when they received either LF diet or TF diet (Figure 4A and 4B). Insulin resistance, as measured by HOMA-IR was significantly higher in TLR4 mutant mice compared to WT mice when received TF diet (11.2 vs. 1.77; P < 0.001) (Figure 4C).

**Figure 5:**
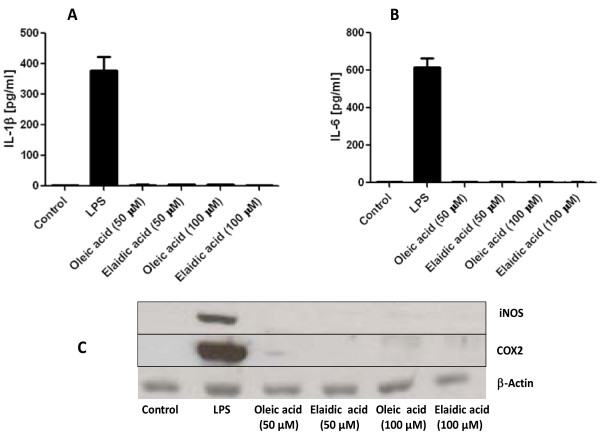
**In vitro experiments on macrophage cell lines (RAW 264.7 and J774A1)**. No upregulation of inflammatory markers such as IL-1β (A), IL-6 (B), COX_2 _(C), or iNOS (C) was observed in macrophages in response to treatment with either elaidic acid or oleic acid at 50 μM and 100 μM concentration level. β-actin was used as a loading control. Abbreviations: COX2, cyclooxygenase 2; iNOS, inducible nitric oxide synthase.

*In vitro *studies in the mouse macrophage cell lines RAW 264.7 and J774A1 showed that there was no upregulation of COX-2 and iNOS by TF. In addition, these cells failed to induce and IL-1β and IL-6 in supernatant in response to macrophages treated with either elaidic acid or oleic acid (Figure 5A-C).

**Figure 6:**
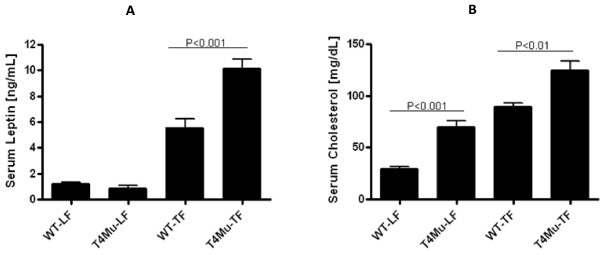
**5-hour fasting serum leptin (A) and serum cholesterol (B) concentrations of WT and T4Mu mice that received LF and TF diets**. Significant difference was determined with 2-way analysis of variance (genotype × diet type) for serum leptin and serum cholesterol. Serum leptin and cholesterol are significantly higher in T4Mu mice compared to WT mice that received TF diet. Serum cholesterol is significantly higher in T4Mu mice compared to WT mice that received LF diet. Abbreviations: LF, low fat diet (13.5% energy from fat); T4Mu, toll-like receptor-4 loss of function mutant mice; TF, trans fat diet (60% of energy from fat, shortening-based); WT, wild type mice.

We measured serum leptin, adiponectin, serum amyloid A, keratinocyte-derived chemokine, and lipocalin-2 concentrations because these have a role in insulin resistance, type-2 diabetes, and obesity. In both genotypes, serum leptin concentrations were significantly higher in mice that received TF diet compared to those that received LF diet (P < 0.001). The mice with loss of function mutation in TLR4 had significantly higher (~82%) serum leptin concentrations compared to WT mice when they received TF diet (P < 0.002) (Figure 6A). However, there was no significant difference in serum concentrations of adiponectin, serum amyloid A, lipocalin-2, and keratinocyte-derived chemokine between genotypes or diet types (data not shown).

### Serum lipids

Serum total cholesterol concentrations were significantly higher in mice that received TF diet compared to those that received LF diet in both genotypes (P < 0.0001). These serum total cholesterol increases were ~140% and ~78% in WT mice and in mice with loss of function mutation in TLR4, respectively. Mice with loss of function mutation in TLR4 had significantly higher total serum cholesterol compared to WT mice regardless of the diet type they received (P < 0.01). Mice with loss of function mutation in TLR4 had ~140% higher total serum cholesterol when they received LF diet and had ~39% higher total serum cholesterol when they received TF diet (Figure 6B). However, serum trigacylglycerol concentrations were not significantly different between either genotype or diet type (data not shown).

## Discussion

Emerging research has established a connection between low grade systemic inflammation and insulin resistance. Insulin resistance is the primary characteristic of obesity and is a risk factor for development of type-2 diabetes. It has been reported that TF intake increased the production of inflammatory markers such as C-reactive protein (CRP), tumor necrosis factor-α (TNF-α), interleukin-6 (IL-6), and E-selectin [2,5,6]. It is likely that TFs are incorporated into plasma membranes of endothelial cells, manocyte/macrophages, and adipocytes which in turn transduces membrane signaling pathways related to inflammation [6]. Although, proinflammatory effect of diets high in TF has been established, the exact molecular mechanism linking TF with inflammatory markers is largely unknown. In this present study, we investigated whether feeding TLR4 mutant mice a diet high in TF would offer protection against TF-induced obesity, insulin resistance, and inflammation in TLR4 mutant mice. We found that loss of function mutation in TLR4 did not protect mice from high TF-diet-induced obesity, hyperglycemia, hyperinsulinemia, hypercholesterolemia, and hyperleptinemia.

What is interesting in this study is that mice with loss of function mutation in TLR4 had gained ~26.4% more weight, had ~39.9% higher blood glucose, had ~57.6% higher serum insulin, had ~78% higher serum cholesterol, and had ~82.5% higher serum leptin compared to their counterpart WT mice when they were fed a diet high in TF. Additionally, TLR4 mutant mice had higher fat deposition than WT mice in the liver when they received TF diet. These observations suggest that functional TLR4 is important in protecting mice from trans fat-induced obesity, hypercholesterolemia, hyperleptinemia, hyperglycemia, and hyperinsulienmia. The exact mechanism through which the loss of function mutation in TLR4 induces the above described pathologies is not known. Recently, our collaborators, Vijay-Kumar et al [24] reported that TLR5 deficient mice also developed obesity, hyperlipidemia, and insulin resistance in response to high fat diet and the transfer of gut microbiota from TLR5 deficient mice to WT-germ-free mice conferred obesity, hyperlipidemia, and insulin resistance (features of metabolic syndrome) to the recipients suggesting that the gut microbiota mediate metabolic derangements associated with high fat diet. Insulin resistance we observed in TLR4 mutant mice in response to TF diet is likely secondary to the increased adiposity of the TLR4 mutant mice. Therefore, it is difficult to accurately assess the effect of TLR4 (or lack of TLR4) on insulin resistance/inflammation in a model where adiposity is not equal.

The possible explanations for the observed results include increased fat absorption in TLR4 mutant mice compared to WT mice or increased energy expenditure in WT mice compared to TLR4 mutant mice may be responsible for the observed pathologies. Also, it is likely that the diet high in TF may have altered the microflora in cecum which in turn may have altered the intestinal barrier allowing translocation of microbial products leading to the activation of TLR4 independent pathways associated with inflammation, dyslipidemia, hyperinsulinemia, insulin resistance, and hyperleptinemia. These pathologies were further exacerbated in the presence of loss of function TLR4 mutation.

To our knowledge, this is the first study that investigated the effects of diet high in TF on obesity and circulating glucose, insulin, leptin, and inflammatory markers in mice with loss of function mutation in TLR4. It has been well established that activation of TLR4 by microbial endotoxin, LPS (TLR4 agonist), triggers the host's innate immune and inflammatory responses leading to the upregulation of pathways relating to insulin resistance and dyslipidemia. One possible mechanism is that LPS stimulates the TLR4 and ERK1/2 signaling which in turn activates hormone sensitive lipase and adipocyte triglyceride lipase. This in turn increases lipolysis in adipocytes leading to free fatty acid (FFA) efflux from adipocytes into the blood [25]. These elevated FFAs interfere with insulin function (unable to suppress hepatic glucose production and stimulate glucose influx into muscle and adipocytes) leading to insulin resistance [26-29]. Shi et al [9] reported that FFAs are themselves capable of triggering TLR4 signaling by trasducing production of proinflammatory markers in macrophages, adipocytes, and liver leading to insulin resistance. Insulin resistance is a main pathological abnormality associated with metabolic syndrome, obesity, and type-2 diabetes. Conversely, loss of function mutation in TLR4 protected against diet-induced obesity, hyperinsulinemia and inflammation in response to diet high in saturated fat [23]. Several other investigators proposed that saturated fatty acids act as agonist for TLR4 and therefore linking dietary fat with innate immune system and inflammation [15-21,30,31]. However, our findings in this model are at odds with the linkage of TLR4 and diet-induced obesity, hyperinsulinemia, and inflammation in response to diet high in saturated fat [23].

Although, we did not observe the blunting of TF diet-induced weight gain, hyperinsulinemina, and hyperglycemia in mice with loss of function mutation in TLR4, our results in this study do not directly contradict the previously proposed connection between saturated fatty acids and upregulation of TRL4 down-stream signaling leading to insulin resistance and inflammation. Well controlled studies by several investigators [23,32] offered strong evidence that saturated fat diet-induced insulin resistance was blunted in mice that lacked functional TLR4. It is likely that saturated fat and trans fat induce inflammation and insulin resistance by all together different mechanisms.

## Conclusions

Our findings suggest that diets high in TF induce obesity, hyperglycemia, insulin resistance, hypercholesterolemia, and hyperleptinemia even in the absence of functional TLR4. Additionally, these pathologies associated with TF diet were exacerbated in the presence of loss of function mutation in TLR4. Furthermore, our results indicate that TLR4 independent pathways may be involved in TF-diet-induced obesity, hyperglycemia, hyperinsulinemia, and hyperleptinemia. Further studies are needed to decipher the complex biomolecular mechanism linking consumption of diets rich in TF with pathologies associated with TLR4 signaling such as insulin resistance, metabolic syndrome, and type-2 diabetes.

## Competing interests

The authors declare that they have no competing interests.

## Authors' contributions

VG, VKM, ATG, and TRG designed research. VKM, JDA, and FAC conducted research. VG analyzed data. VG obtained funding for the study. VG wrote and revised the manuscript. VG, VKM, JDA, FAC, TRZ, and ATG read and approved the final manuscript.
